# *Ab initio* study of electronic, elastic, thermodynamic, photocatalytic properties of double antiperovskite, Cs_6_AgBiX_2_ (X = Cl, Br, I)

**DOI:** 10.1039/d4ra05661b

**Published:** 2024-11-05

**Authors:** Laraib Sajid, M. Usman Saeed, S. H. Mashadi, S. Sheryar Abid, Shamiala Pervaiz, Zeeshan Ali, Yousef Mohammed Alanazi, Aziz-Ur-Rahim Bacha, Y. Saeed

**Affiliations:** a Department of Physics, Abbottabad University of Science and Technology Abbottabad KPK Pakistan yasir.saeed@kaust.edu.sa yasirsaeedphy@aust.edu.pk +(92)-3454041865; b Department of Computer Sciences, Abbottabad University of Science and Technology Abbottabad KPK Pakistan; c Department of Electrical Engineering, University of Azad Jammu & Kashmir Muzaffarabad Pakistan; d College of Engineering, Chemical Engineering Department, King Saud University Riyadh Saudi Arabia; e State Key Laboratory of Urban Water Resource and Environment, Shenzhen Key Laboratory of Organic Pollution Prevention and Control, School of Civil and Environmental Engineering, Harbin Institute of Technology Shenzhen Shenzhen 518055 P. R. China

## Abstract

In this paper, we use density functional theory (DFT) using full-potential linearized augmented plan wave plus local orbital method (FP-LAPW + lo). The structural, electronic, optical, photocatalytic, mechanical, vibrational, and thermodynamical behaviors of new double antiperovskite (DAP) Cs_6_AgBiX_2_ (X = Cl, Br, I) were studied. The band structure was calculated with and without spin orbit coupling (SOC). Using the TB-mBJ approach (Hybrid) revealed bandgap values of 1.504 eV, 1.491 eV, and 1.392 eV for Cs_6_AgBiCl_2_, Cs_6_AgBiBr_2_, and Cs_6_AgBiI_2_, respectively. Optical characteristics were studied to ascertain the light absorbing ability of Cs_6_AgBiX_2_. The elastic and vibrational (phonon) properties demonstrate that Cs_6_AgBiCl_2_ and Cs_6_AgBiBr_2_ are stable but Cs_6_AgBiI_2_ is not. The calculated optimal bandgap and high absorption coefficient of Cs_6_AgBiCl_2_ and Cs_6_AgBiBr_2_, suggest their potential for solar cell applications. Moreover, our photocatalytic results suggest that these materials have high oxidizing capacity that can be used to efficiently produce oxygen cheaply using solar water splitting.

## Abstract 2

We use DFT to study the structural, electronic, optical, photocatalytic, mechanical, vibrational, and thermodynamical behaviors of new double antiperovskite Cs_6_AgBiX_2_ (X = Cl, Br, I).

## Graphical abstract

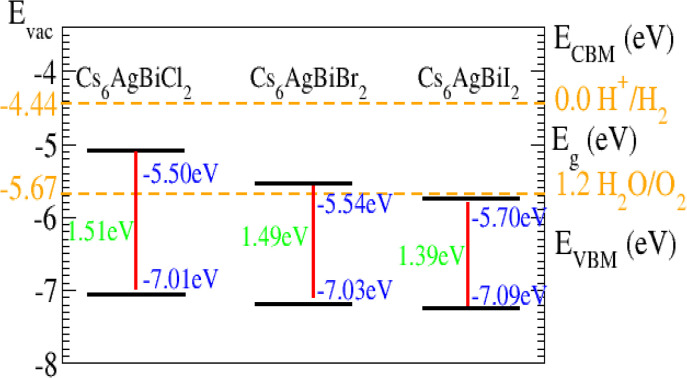

## 1 Introduction

Growing industrialization and fossil fuel use has significantly contaminated the environment and there is now a global energy crisis. To address this, researchers are turning to renewable energy sources, with solar energy being a key focus due to its safety and eco-friendliness.^1–3^ Perovskite materials are gaining attention for solar energy use because of their great properties like effective light absorption and improving power conversion efficiency. Researchers have been studying these materials extensively.^4–6^

Perovskite has the crystal structure CaTiO_3_ and the formula ABX_3_, it is used in different forms like antiperovskite X_3_BA and double perovskite A_2_B′B′′X_6_. These structures offer unique qualities, like easy synthesis and stability.^7–9^ A recent addition is the double antiperovskite X_6_B′′B′A_2_, showing promise for various applications.^10^ Hybrid halide perovskites, like CH_3_NH_3_PbX_3_ are widely studied for efficient solar cells but their sensitivity to moisture and use of toxic lead, pose challenges. Seeking stable, lead-free alternatives has led to the exploration of double perovskite halides, such as Cs_2_AgBiX_6_. These compounds, like Cs_2_AgBiCl_6_ and Cs_2_AgBiBr_6_, are non-toxic and stable, making them promising for solar applications compared to the lead-containing alternatives. The unique structure of halide double perovskites has gained attention as a potential replacement for lead-based perovskite materials.^11–15^

van der Waals (vdW) interactions have been shown to have a significant impact on the geometry optimization and electronic properties of inorganic CH_3_NH_3_(Pb,Sn)(I, Br, Cl)_3_ halide perovskites. Studies have focused on the effects of these interactions on the structural and electronic properties of these perovskites. A hydrogen/ionic link between the halogen atoms and an ordinary amine group (NH_3_^+^) creates the bonding between organic and inorganic components (van der Waals interaction).^16,17^ Wang *et al.*^18^ presented a PD with a hybrid vdW heterostructure made of graphene (Gr)/1D CH_3_NH_3_PbI_3_ and hexagonal boron nitride (h-BN).

However, vdW heterostructures based on double perovskites are still uncommon but are known to have a significant role on the properties of 2D perovskites. Gao *et al.* constructed different heterostructures made up of CsCl, NaInCl, CsNaInCl, and Cl interfaces of the inorganic double perovskite Cs_2_NaInCl_6_ and two-dimensional transition metal–sulfur complexes XS2 (X = Cr, Mo, and W).^19^ Using DFT calculations, properties of the 2D (C_4_H_9_NH_3_)_2_PbI_4_/black phosphorus (BP) vdW heterostructure have been investigated.^20^ Furthermore, density functional theory considering vdW, was used to calculate the electron structure and interface properties of inorganic perovskite (without organic cation), results show weak vdW forces between the interfaces of Cs_2_AgBiBr_6_ and Ti_3_C_2_T_*x*_ (to maintain its semiconductor characteristics).^21^ There is no study for vdW interactions in 3D inorganic double perovskites or double anti-perovskites as compared to 2D and heterostructures.

Recently, Rani *et al.* explored some new double antiperovskites Na_6_SOCl_2_, Na_6_SOBr_2_, Na_6_SOI_2_, K_6_SOCl_2_, K_6_SOBr_2_ and K_6_SOI_2_ with bandgaps of 4.34 eV, 3.71 eV, 3.33 eV, 3.99 eV, 3.38 eV and 2.90 eV, respectively, and show that they are good for thermoelectric applications.^22^ Yu *et al.* predict that Na_6_SOI_2_ is specifically appealing for solid sodium-ion battery applications at low temperatures.^23^ Mebrouki *et al.* examined the process by which temperature effects the BaVO_3_ elastic constants *C*_11_, *C*_12_, and *C*_44_, less progressively as temperature rises.^24^ Djebari *et al.* calculated the octahedral factor, the formation energy, the tolerance factor *etc.* to confirm the structural stability of A^3+^B^4+^(O_2_N)^7−^ and A^2+^B^5+^(O_2_N)^7−^. Based on the computed bandgaps, photovoltaic and ferroelectric devices could find use for them.^25^ Gao *et al.* discovered that the coefficient of thermal expansion is negatively impacted by variations in pressure and temperature.^26^ Since the double antiperovskite has just recently been proposed, there are not many reports on tests or theoretical calculations, suggesting that this might be a new line of inquiry. Presently, we are examining the features of double antiperovskites Cs_2_AgBiX_6_ (X = Cl, Br, I). We are examining the expected material’s structural, electrical, elastic and optical properties, focusing on those with a bandgap similar to MAPI.^27^

## 2 Computational details

Using the Wien2k code, we ran our calculations using the full-potential linearized augmented plane wave plus local orbitals (FP-LAPW + lo) technique, which is a component of DFT.^28^ Using the more accurate exchange-correlation potential flavor of the generalized gradient approximation (WC-GGA), structural, electrical, and thermodynamic features are investigated.^29^ To acquire precise bandgap data, we implemented the modified Becke-Johnson (mBJ) scheme across the WC-GGA.^30,31^ In the past, the mBJ plan provided promising band structures and bandgap values for lead halide perovskites and other semiconductors when compared to experimental results.^32–37^ The scheme mBJ across WC-GGA gives results close to the experimental results for Cs_6_AgBiX_2_ (X = Cl, Br and I). The accuracy of our calculations is mainly affected by two factors: one is the total number of *k* points, so for the Brillouin zone (BZ) integration a *k*-mesh of 13 × 13 × 13 was used and a finer *k*-mesh of 24 × 24 × 24 for electronic and optical properties. Another factor is cutoff kinetic energy which determines the number of plane wave expansions inside the muffin-tin radii, here the cutoff energy is set to be −7.0 Ryd, which separates the valence states from the core states of electrons. The plane-wave expansion cutoff was expanded up to *G*_max_ = 20 and the angular momentum of the sphere is *l*_max_ = 12. All structures were optimized with an energy convergence tolerance of up to 10^−5^ Ryd.^38–41^

## 3 Results and discussion

### 3.1 Structural properties

**Fig. 1:**
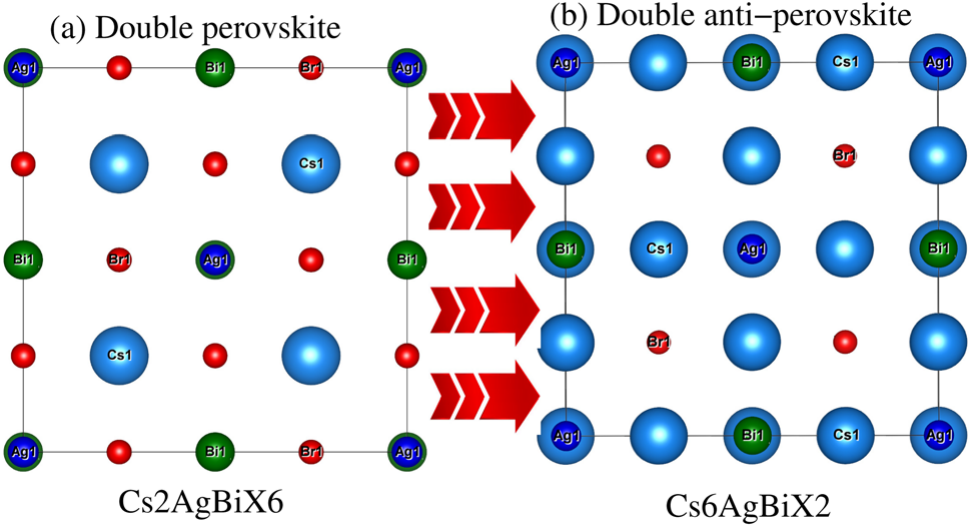
Prospective view of (a) double perovskite and (b) double anti-perovskite crystal structures.

**Fig. 2:**
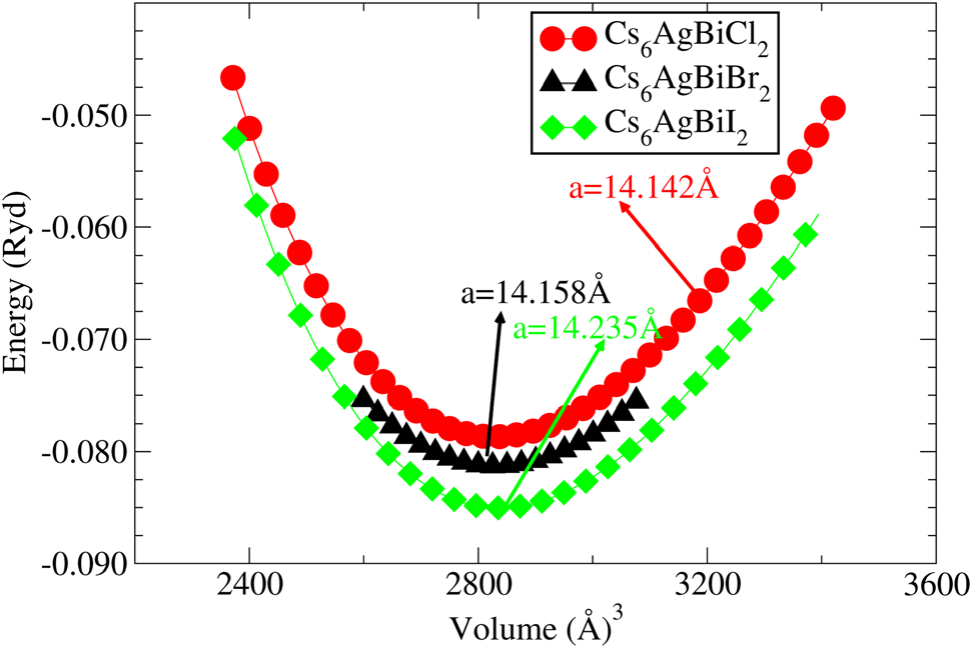
For cubic double anti-perovskites, the energy *vs.* volume curve, Cs_6_AgBiX_2_ (X = Cl, Br, I).

Understanding the different physical characteristics of a substance is crucial and understanding structural characteristics is essential for this. Fig. 1 shows the transformation of double perovskite into a double anti-perovskite crystal structure. The proposal aims to optimize the structural properties of Cs_6_AgBiX_2_ (X = Cl, Br, I) by reducing the energy to its ground state. The starting point for the optimization procedure is the lattice parameter of experimental Cs_2_AgBiCl_6_. The volume optimization curve, which represents the relationship between energy and volume, is plotted in Fig. 2 for Cs_6_AgBiX_2_ (X = Cl, Br, I). To fit the nonlinear plot of energy *vs.* volume we used the initial lattice parameter values for Cs_2_AgBiX_6_ (X = Cl and Br) as a reference. The volume optimization was used to calculate structural parameters such as bulk modulus *B* (GPa) and lattice constant *a* (Å), for Cs_6_AgBiX_2_ (X = Cl, Br, I). Table 1 shows the structural characteristics, which were determined by applying Birch–Murnaghan's equation of states.^44^ The optimized lattice constants for all compounds fall within the range 14.14 Å to 14.24 Å, closely aligning with experimentally synthesized double perovskites like Cs_2_AgBiCl_6_ and Cs_2_AgBiBr_6_, which have lattice constants of 10.77 Å and 11.27 Å.^14^ The optimized bulk modulus (*B*) ranges from 4.63 GPa to 5.16 GPa as presented in Table 1. Unfortunately, experimental or theoretical data for comparison with these optimized compounds is not available.

**Table 1:** Optimized lattice parameters, bulk modulus and bandgap of double antiperovskites Cs_6_AgBiX_2_ (X = Cl, Br, I)

Materials	*a* = *b* = *c* (Å)	Bulk modulus *B* (GPa)	GGA *E*_g_ (eV)	GGA + SOC *E*_g_ (eV)	Hybrid *E*_g_ (eV)
Cs_6_AgBiCl_2_	14.14	4.63	0.91	0.72	1.50
Cs_6_AgBiBr_2_	14.16	5.10	0.84	0.65	1.49
Cs_6_AgBiI_2_	14.24	5.16	0.78	0.59	1.39

### 3.2 Electronic band structure

**Fig. 3:**
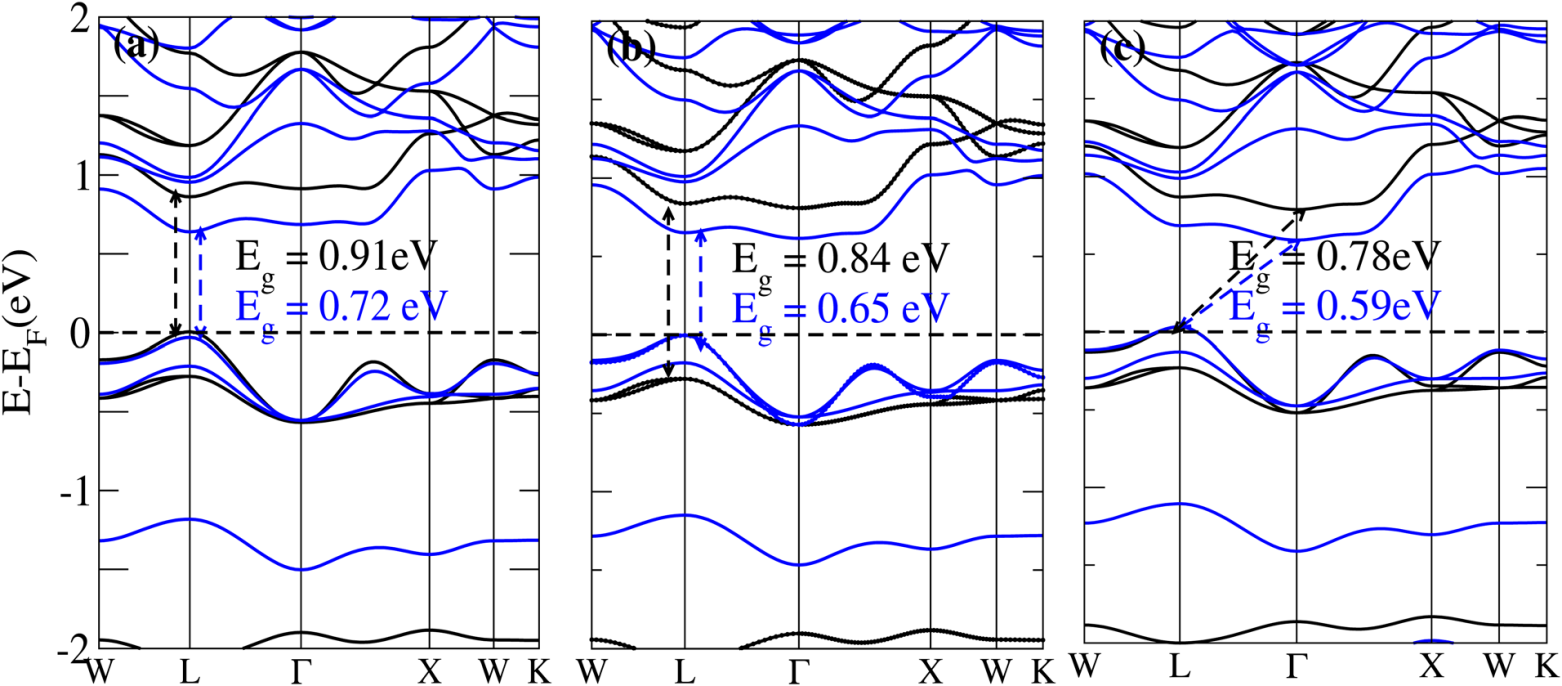
The band structure of Cs_6_AgBiX_2_ (X = Cl, Br, I) without SOC (black) and with SOC (blue).

**Fig. 4:**
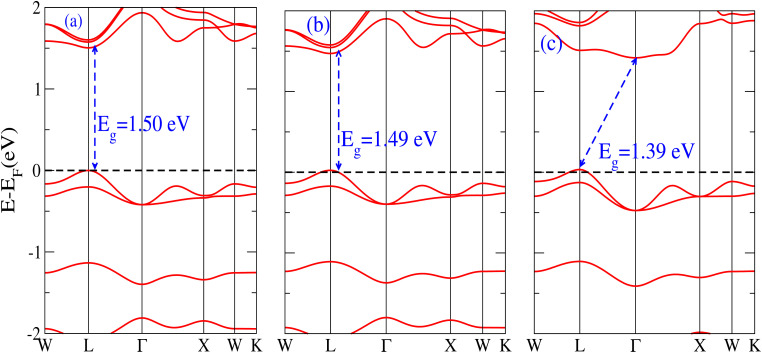
The band structure of Cs_6_AgBiX_2_ (X = Cl, Br, I) with hybrid.

The behavior of a material is profoundly shaped by its band structure, a critical aspect for studying its properties. Analyzing the band structure, including determining its metallic or semiconducting nature, provides valuable insights into the electronic characteristics of the material. This electronic band structure is essential for understanding the optical behaviour and resistivity of solids, which are important for the design of solid-state electronics like solar cells and transistors. The band gap is either direct or indirect, the dispersion of bands informs us of the electronic behavior through which we can specify the physical properties of materials. Consequently, we conducted band structure calculations for Cs_6_AgBiX_2_ (X = Cl, Br, I) and examined their electronic conduct for further analysis. The initial bandgap calculations for each compound utilized a simple self-consistent field (SCF) approach without considering spin–orbit coupling (SOC). Similarly, this was also done in order to obtain bandgap values that nearly match the experimental bandgap value of MAPI (1.55 eV), producing a straight bandgap as a result at the symmetry point similar to MAPI.^42^ Additionally, mBJ + SOC (Hybrid) was utilized to modify the bandgap values in order to better correlate with the experimental MAPI, as a good solar cell absorber. The bandgap of CH_3_NH_3_PbCl_3_ is 3 eV, while the band gap of CH_3_NH_3_PbBr_3_ is 2.26 eV.^43^ The experimental (theoretical) bandgap values for Cs_2_AgBiCl_6_ and Cs_2_AgBiBr_6_, which were previously reported as 2.77 (2.62) eV and 2.19 (2.06) eV, respectively, were recalculated as a baseline with a similar approach.^14,43^ We computed bandgap values of 1.50 eV (Cl), 1.49 eV (Br), and 1.39 eV (I) for Cs_6_AgBiX_2_ (X = Cl, Br, I) with hybrid, respectively. These are close to MAPI, hence proving that anti-double perovskite could reduce the bandgap values substantially as compared to double perovskites. While with SOC, and without SOC, bandgap results are listed in Table 1 and are explained in Fig. 3 and 4.

Our analysis leads to the conclusion that the FP-LAPW + lo technique, without considering mBJ + SOC, tends to substantially underestimate bandgap values in double antiperovskites. Consequently, our objective is to identify a lead-free material with a bandgap value approximately equal to 1.5 eV, serving as a potential replacement for MAPI at room temperature.^45^

The bandgap values for Cs_6_AgBiCl_2_, Cs_6_AgBiBr_2_ and Cs_6_AgBiI_2_ are 0.91 eV, 0.84 eV and 0.78 eV, in the absence of SOC. The bandgap values of these materials with and without SOC are depicted in Fig. 3a–c. The band structure of Cs_6_AgBiCl_2_ has a bandgap value of 0.91 eV in the absence of SOC and 0.72 eV in the presence of SOC. The band structure of Cs_6_AgBiBr_2_ has a bandgap value of 0.84 eV in the absence of SOC and 0.65 eV in the presence of SOC. These two materials have a direct bandgap as shown in Fig. 3a and b while the band structure of Cs_6_AgBiI_2_ has a bandgap value of 0.78 eV in the absence of SOC and 0.59 eV in the presence of SOC as shown in Fig. 3c.

It is observed that the bandgap values decreased slightly for Cs_6_AgBiX_2_ (X = Cl, Br, I) after applying SOC. The bandgap values of Cs_6_AgBiCl_2_ and Cs_6_AgBiBr_2_ align closely with the experimentally calculated values of CH_3_NH_3_PbI_3_ (1.55 eV). Within the bandgap of 1.39 to 1.50 eV (see Fig. 4), efficient semiconductor solar cells can be developed, enabling the release of electrons without generating a substantial amount of heat. While some recently investigated double antiperovskites have a direct bandgap, their values exceed this optimal range. Others within the mentioned range, although having an indirect bandgap, are not suitable for solar cell applications. Pressure-induced bandgap tuning could potentially bring the bandgap close to 1.5 eV.

### 3.3 Density of states (DOS)

**Fig. 5:**
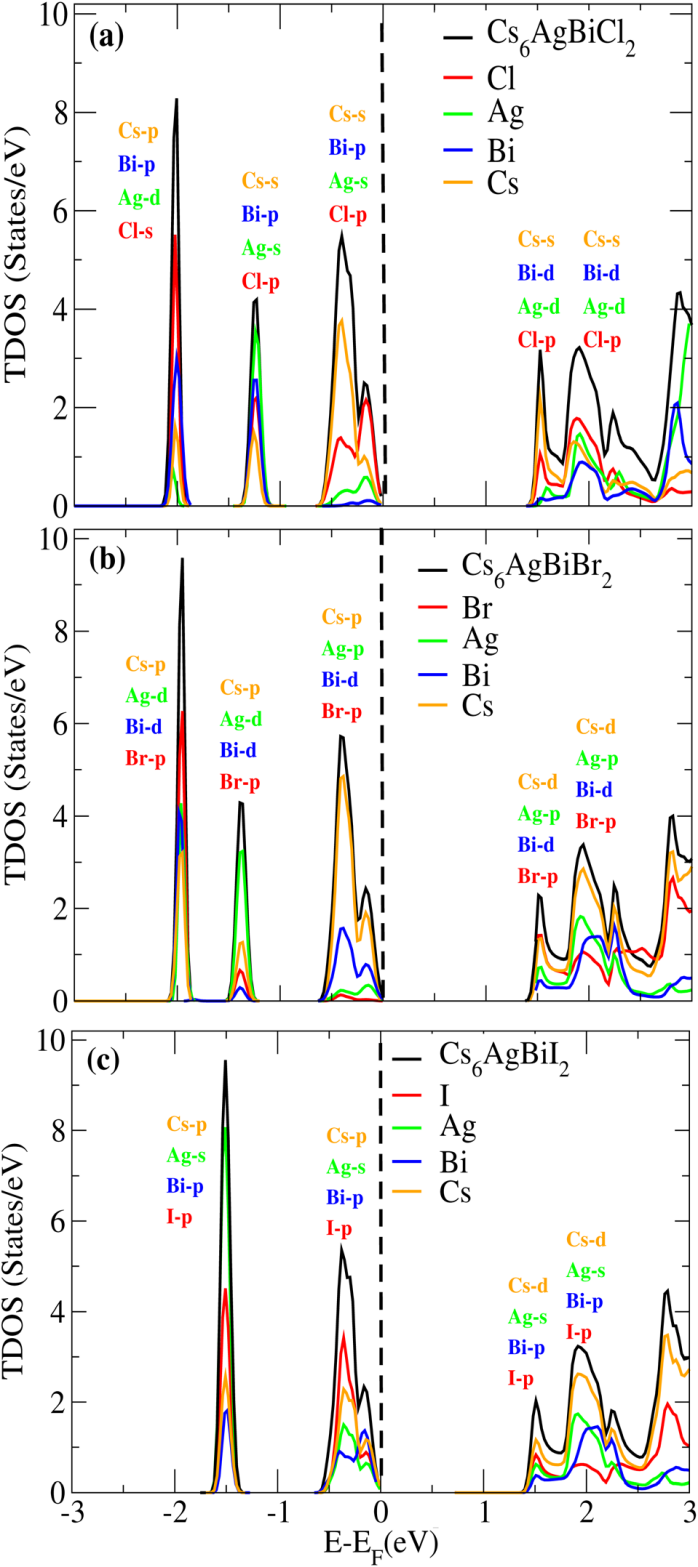
Density of states (DOS) of (a) Cs_6_AgBiCl_2_, (b) Cs_6_AgBiBr_2_ and (c) Cs_6_AgBiI_2_.

We examined the density of states (DOS) for Cs_6_AgBiX_2_ (X = Cl, Br, I) in order to evaluate the individual contributions of Cs, Ag, Bi, Cl, Br and I atoms to the valence band maxima (VBM) and conduction band minima (CBM), as depicted in Fig. 5a–c.

In Fig. 5a and b, it is evident that the VBM is predominantly influenced by the Cs atom in both materials, while the CBM is shared between Cs and Cl/Br atoms. Notably, Br atoms significantly contribute to the bandgap value. Further insights into the orbital contributions of Cl and Br in the VBM and CBM were gained by examining the PDOS, which is also illustrated in Fig. 5 for Cs_6_AgBiX_2_ (Cl, Br, I).

The range of the valence band is −3 to 0 and the conduction band range is 0 to 3, and the 0 point represents the Fermi level. Fig. 5a reveals that the Cs-s orbital plays a dominant role in both CBM and VBM, while other atoms have a relatively small contribution compared to these two atoms. The p-orbital of Cl also exhibits significance in the CBM, whereas the contributions of d orbitals are minor in VBM. In VBM, the p-orbital of Bi plays a crucial role, with Cl's p-orbital occupying the entire VBM up to 2 eV, however Br's d-orbital does not contribute much to either the CBM or VBM. Also, compared to Ag and Bi, the effect of Cl's p-orbital on VBM is greater. According to Fig. 5b, comparable patterns are seen in the DOS: Br's p-orbital and Cs's d-orbital completely dominate the CBM and Cs's p-orbital completely dominates the VBM. According to Fig. 5c, similar patterns are seen in the DOS: I's p-orbital and Cs's p-orbital completely dominate the CBM and Cs's d-orbital completely dominates the VBM.

### 3.4 Optical properties

Studying the optical characteristics of materials is essential from the standpoint of the industrial manufacture of optical devices like sensors, lasers, modulators, solar calculators and optical coatings. One promising option for optical materials production is perovskite high-performing optoelectronic devices.^45,46^ The optical characteristics such as the complex dielectric function, *ε*(*ω*) = *ε*_1_(*ω*) + *iε*_2_(*ω*), can be utilized to describe a material's reaction to an external electromagnetic field,^47^ where *ε*_1_(*ω*) and *ε*_2_(*ω*) are the real and imaginary parts of *ε*(*ω*). In the relation *ε*_1_(*ω*), is the dielectric constant, which expresses the amount of material polarized by induced electric dipole formation in the presence of an electric field, whereas *ε*_2_(*ω*) indicates the capacity for electromagnetic waves to transmit and attenuate them. The dielectric characteristics *ε*(*ω*) are closely linked to the combined density of states (DOS) and transition momentum matrix components. The real part of the dielectric function, *ε*_1_(*ω*), can be obtained from *ε*_2_(*ω*) using Kramers–Kronig relationship.^48^ The wave is diminishing and cannot propagate if the value of *ε* < 0; if *ε*_1_(*ω*) > 0 light can propagate through the material.^49,50^

The dispersion of the real and imaginary components of the dielectric characteristics can be analyzed to compute the refractive index, optical conductivity, reflection and absorption coefficients among other optical properties.

The optical properties of solids can be explained by the refractive index *n*(*ω*) and extinction co-efficients *k*(*ω*). In the case of *n*(*ω*) < *k*(*ω*) the incident rays are reflected and the behavior of materials is dielectric to metallic. It is more advantageous for materials with high dielectric constants to be used in solar cells since they often have low exciton binding energy.^51^

**Fig. 6:**
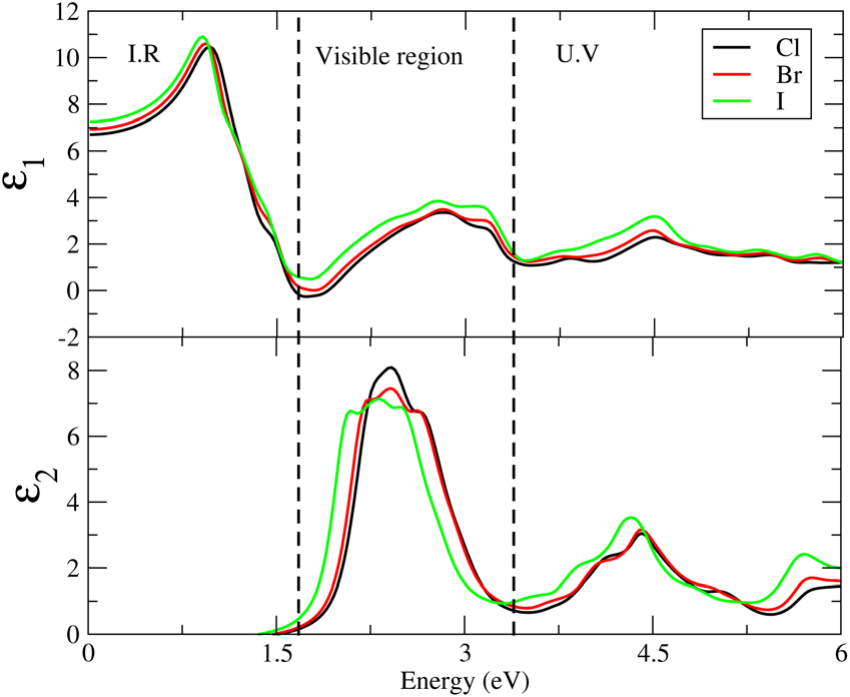
The dielectric constants for Cs_6_AgBiX_2_ (X = Cl, Br, I).

**Fig. 7:**
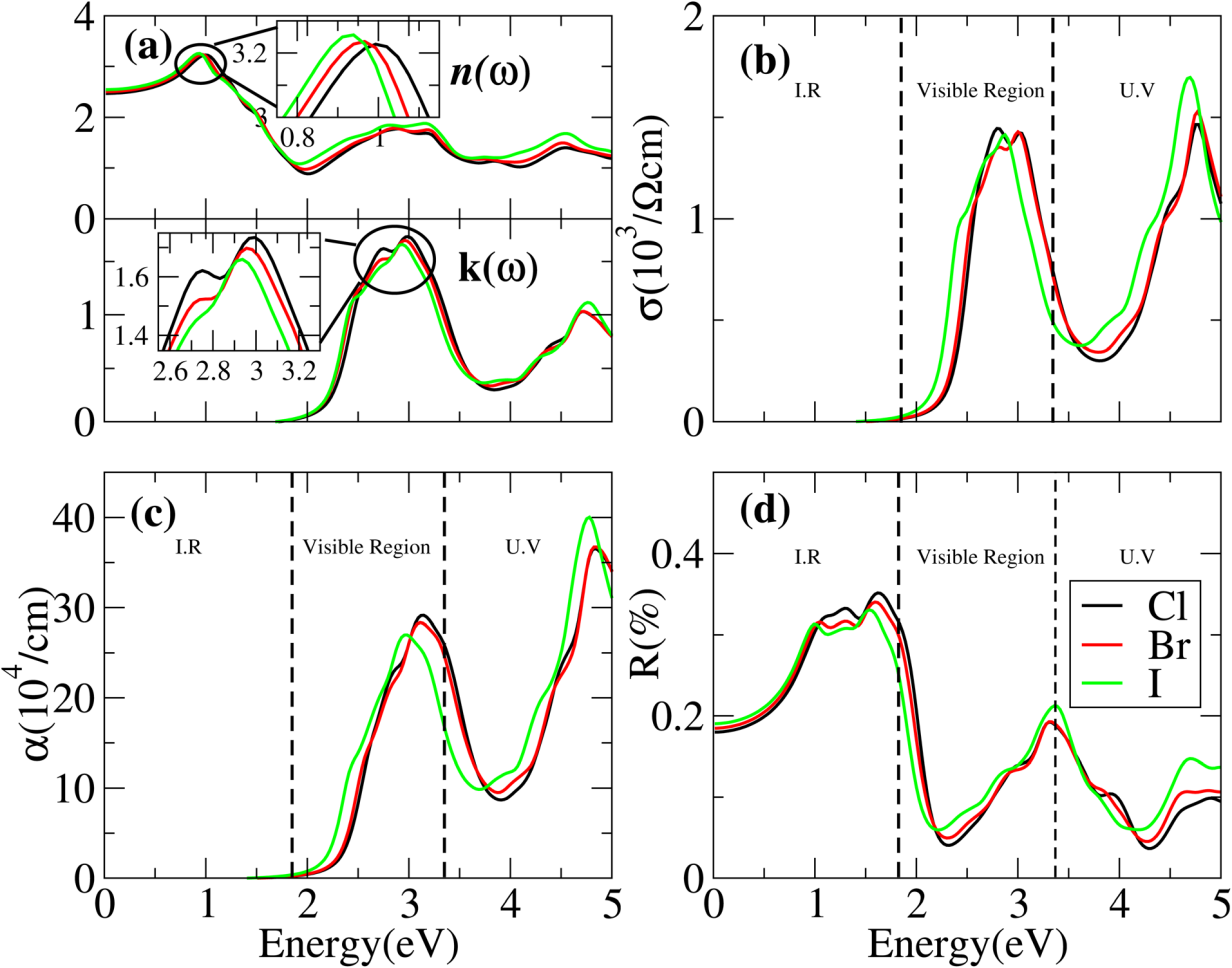
Optical properties such as (a) refractive index, *n* and extinction coefficient, *k*, (b) optical conductivity, *σ*, (c) absorption coefficient, *α*, (d) reflectivity, *R* of Cs_6_AgBiCl_2_, Cs_6_AgBiBr_2_ and Cs_6_AgBiI_2_.

Cs_6_AgBiX_2_ (Cl, Br, I) have *ε*_1_ 9.57, 9.75 and 10.19, respectively, which are greater than Cs_2_LiTlBr_6_ (1.6), Cs_2_NaTlBr_6_ (1.7), Cs_2_AgCrI_6_ (5.7), MAPbI_3_ (5.4) and FAPbI_3_ (5.7), other potential candidates for solar cell applications.^51–56^ It is important to reach maximum polarization characteristics, as indicated by the size of *ε*_1_(*ω*), in order to obtain optimal semiconductor performance. In the visible light range, *ε*_1_(*ω*) approaches peak polarization values of 11.96, 8.77 and 8.63 at 1.0 eV, 0.91 eV, and 0.90 eV, respectively; and *ε*_2_(*ω*) imaginary part shows the light absorption with *ε*_2_(*ω*) values 8.6, 7.9 and 7.5 at −3.0 eV, −3.8 eV, −4.3 eV. None of these compounds are in the visible region. Computations based on the distribution of the dielectric characteristics with real and imaginary parts are shown in Fig. 6, and provide important new information on the linear optical behavior of these materials. We calculated parameters such as the refractive index (*n*), extinction coefficient (*k*), absorption coefficient *I*(*ω*), optical conductivity *σ*(*τ*), and reflectivity *R*(*ω*), in order to analyze the optical properties of Cs_6_AgBiX_2_ (X = Cl, Br, I), as illustrated in Fig. 7. For Cs_2_LiTlBr_6_ and Cs_2_NaTlBr_6_, the static dielectric constants *ε*(0) are 1.6 eV and 1.7 eV, respectively.^46^ So here our study produced comparable findings. Looking at Fig. 7a, we can see that both materials, Cs_6_AgBiCl_2_ and Cs_6_AgBiBr_2_, have a static refractive index (*n*(*ω*)) with values of 3.2 and 3.1, respectively. In Cs_2_LiTlBr_6_ the highest values for *n*(*ω*) and *k*(*ω*) are 1.8 eV and 2.6 eV, while for Cs_2_NaTlBr_6_, these values are 2.5 eV for *n*(*ω*) and 3.2 eV for *k*(*ω*). The optical conductivity (*σ*) of Cs_6_AgBiCl_2_ peaks at 650 (Ω cm)^−1^ at 2.6 eV in Fig. 7b, whereas Cs_6_AgBiBr_2_ peaks at 500 (Ω cm)^−1^ at 3 eV. Because of this, Cs_6_AgBiCl_2_ has exceptionally high optical conductivity in the visible light spectrum (1.77 eV to 3.1 eV) which is important for solar cell applications. The efficiency of solar energy conversion is often described by the absorption coefficient, representing how specific light frequencies penetrate the material before absorption. For Cs_6_AgBiCl_2_ and Cs_6_AgBiBr_2_, the absorption coefficient spectrum *I*(*ω*) shows peaks of 12 × 10^4^ cm^−1^ at 2.6 eV (477 nm) and 11 × 10^4^ cm^−1^ at 3.2 eV, respectively (Fig. 7c). These values are comparable to those of MAPI (methylammonium lead iodide) at 10^4^–10^5^ cm^−1^. The absorption coefficient increases rapidly as incident photons approach the absorption edge, with Cs_6_AgBiCl_2_ exhibiting higher absorption in the visible region compared to Cs_6_AgBiBr_2_. Lastly, in Fig. 7d, the maximum reflectivity *R*(*ω*) for Cs_6_AgBiCl_2_ and Cs_6_AgBiBr_2_ is 13.5% at approximately 1.6 eV and 9% at 2.2 eV, respectively.

### 3.5 Photocatalytic properties

Suitable indirect bandgap semiconductors can be used to utilize solar energy to generate hydrogen by dissociating water.^57,58^ Therefore, clean renewable energy can be produced through photocatalytic water splitting.^59,60^ Water is oxidized by holes and reduced by electrons in the photocatalytic process.^61^

The oxidation–reduction potential of O (1.23) eV for this operation needs to be smaller (higher) than the conduction (valence) band photocatalytic water splitting for all materials under consideration (Table 2).

**Table 2:** Photocatalytic parameters of Cs_6_AgBiX_2_ (X = Cl, Br, I)

Compounds	*χ* (eV)	*E*_g_ (eV)	*E*_CBM_	*E*_VBM_
Cs_6_AgBiCl_2_	1.17	1.50	−1.06	−2.57
Cs_6_AgBiBr_2_	1.16	1.49	−1.10	−2.59
Cs_6_AgBiI_2_	1.14	1.39	−1.26	−2.65

Mullikan electronegativity is used to explore this:

1 *E*_VBM_ = *χ* − *E*_elec_ + 0.5*E*_g_

2 *E*_CBM_ = *E*_VBM_ + *E*_g_

**Fig. 8:**
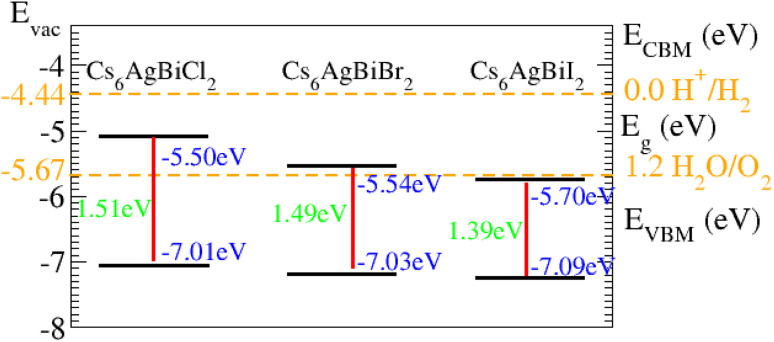
Photocatalytic properties of Cs_6_AgBiCl_2_, Cs_6_AgBiBr_2_ and Cs_6_AgBiI_2_.

As displayed in Fig. 8, on the hydrogen scale the standard oxidation and reduction potentials for photocatalytic water splitting are (−4.44 eV) and (−5.67 eV), respectively.^62^ The Fermi level is set to (−4.44) eV in order to determine the band edge positions of the VB and CB with respect to standard oxidation.^62^ The CB and VB are set to (0 eV = −4.44 eV) and (1.23 eV = ¬5.67 eV).^63^ It is clear from the figure that all the materials show good responses for the oxidation of water but fail to produce hydrogen from water.

### 3.6 Elastic properties

**Fig. 9:**
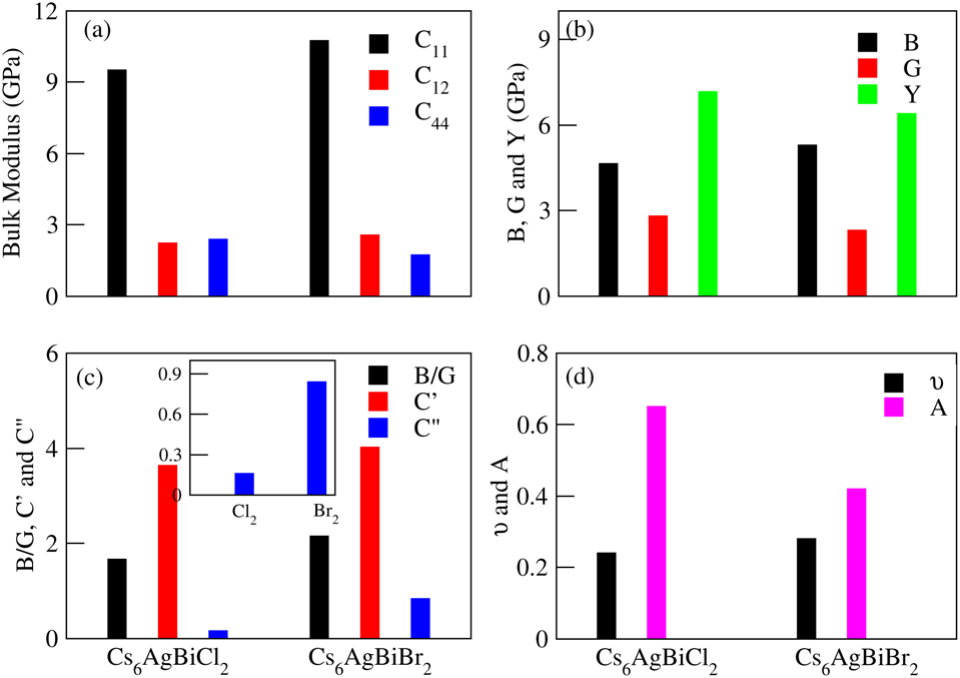
Graphical view of elastic properties for elastically stable Cs_6_AgBiCl_2_, Cs_6_AgBiBr_2_ and Cs_6_AgBiI_2_.

The effective elastic constants are essential for determining a material's practical applications, providing information about its reactivity to outside influences and structural stability. The elastic constants *C*_11_, *C*_12_, *C*_44_, shear moduli *G* and Bulk moduli *B* are the key points of elastic properties. These properties help us to understand the material demand in industrial applications. In the case of Cs_6_AgBix_2_ (x = Cl, Br, I) the calculated elastic constants are detailed in Table 3 and graphical analysis is given in Fig. 9. The materials Cs_6_AgBiCl_2_ are Cs_6_AgBiBr_2_ meet the requirements of Born–Huang stability for cubic crystal stability, that is *C*_11_ > 0, *C*_44_ > 0, *C*_11_ + 2*C*_12_ > 0, *C*_11_ − *C*_12_ > 0 and *C*_12_ < *B* < *C*_11_ are satisfied^64^ while Cs_6_AgBiI_2_ does not meet the criteria. So it is unstable.

**Table 3:** Elastic properties of Cs_6_AgBiX_2_ (X = Cl, Br, I)

Constants	Cs_6_AgBiCl_2_	Cs_6_AgBiBr_2_	Cs_6_AgBiI_2_
*C*_11_	9.5	10.74	9.15
*C*_12_	2.22	2.56	3.17
*C*_44_	2.38	1.72	−3.65
*B*	4.64	5.10	5.16
*G*_v_	2.8	2.67	−0.99
*G*_R_	2.7	2.24	−32.67
*G*_H_	2.8	2.45	−16.83
*Y*	7.17	6.86	−3.18
*B*/*G*	1.66	2.15	0.30
*C*′′	−0.16	0.84	6.82
*ν*	0.24	0.28	0.60
*A*	0.65	0.42	−1.22
*C*′	3.64	4.09	2.99
*A*_z_	1.73	1.55	0.54
*A*_u_	0.22	0.95	−4.84
*ξ*	0.38	0.39	0.49
*T*(*m*)	609.15	616.48	607.08

The elastic constants can be calculated using the following Voigt–Reuss–Hill approximation where, in the formulas *B*_V_ = *B*_R_ = *C*_11_ + 2*C*_12_/3, *G*_V_ = *C*_11_ − *C*_12_ + 3*C*_44_ and *G*_R_ = 5*C*_44_(*C*_11_ − *C*_12_)/(4*C*_44_ + 3*C*_11_ − 3*C*_12_), subscripts V and R stand for Voigt bound and Reuss bound, respectively.^65^ In Table 3, we can see that the *G*_v_ of Cs_6_AgBiX_2_ (Cl, Br, I) decreases continuously which shows that the resistive behavior of these materials is in the order Cs_6_AgBiCl_2_ > Cs_6_AgBiBr_2_ > Cs_6_AgBiI_2_.

Poisson's ratio (*ν*) or Pugh's ratio (*B*/*G*) can be used to evaluate a material's toughness and brittleness. When *ν* is less than 0.26 or *B*/*G* is less than 1.75, the material is considered ductile.^66^ Here, only one material, Cs_6_AgBiCl_2_, is ductile while the other two are brittle as their *ν* and *B*/*G* values are greater than 0.26 and 1.75, respectively. Ionic materials typically have a *ν* range of 0.3–0.4, but the limit for pure ionic materials is 0.5. Covalent materials usually have *ν* values around 0.2 and for Cs_6_AgBiX_2_ (X = Cl, Br, I), they are 0.24, 0.28 and 0.59, indicating a greater ionic property than covalent property.^67^

Elastic anisotropy is associated with the development of microcracks during the use of a material, we computed this for Cs_6_AgBiX_2_ (X = Cl, Br, I) using the anisotropy index *A* = 2*C*_44_/(*C*_11_ − *C*_12_). A material is isotropic if *A* is either 0 or 1; otherwise, it exhibits anisotropy.^68,69^ The mechanical properties, given in Table 3, were analyzed using standard relations. Cs_6_AgBiCl_2_ shows greater shear modulus (*G*_H_) and Young's modulus (*Y*) values, indicating greater stiffness and resistance against plastic deformation compared to Cs_6_AgBiBr_2_.

Cauchy's pressure (*C*′′ = *C*_12_ − *C*_44_) values, whether positive or negative are presented in Table 3, further elucidate the ductile nature of Cs_6_AgBiCl_2_. The positive values of *C*′′ for Cs_6_AgBiBr_2_ and Cs_6_AgBiI_2_ reinforce the materials' brittleness. The shear constant (*G*_H_), determining a material's dynamic stability, has a positive value for Cs_6_AgBiCl_2_, Cs_6_AgBiBr_2_ and Cs_6_AgBiI_2_ in Table 3, affirming their mechanical stability.

**Fig. 10:**
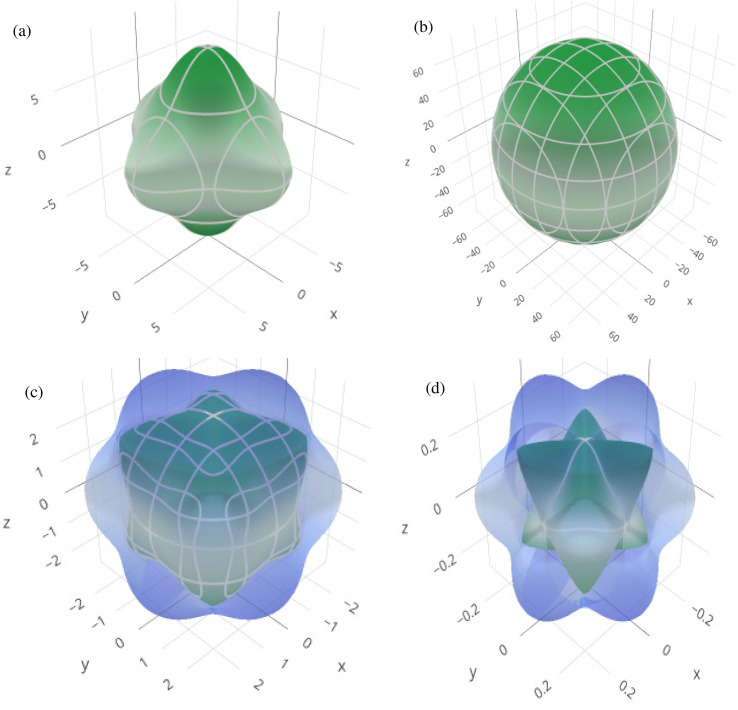
3D spatial dependence of (a) Young's modulus, (b) linear compressibility, (c) shear modulus and (d) Poisson's ratio for Cs_6_AgBiCl_2_.

**Fig. 11:**
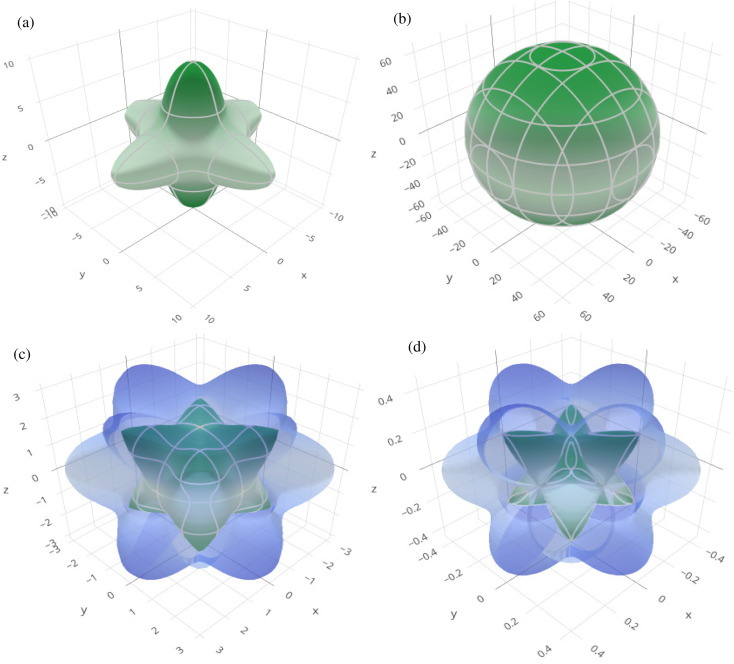
3D spatial dependence of (a) Young's modulus, (b) linear compressibility, (c) shear modulus and (d) Poisson's ratio for Cs_6_AgBiBr_2_.

In order to graphically portray the anisotropic and elastic behavior of the materials under study, three-dimensional (3-D) contour plots of the Young's modulus (*Y*), linear compressibility (*β*), shear modulus (*G*), and Poisson's ratio (*ν*) have been created using the Elate Code.^70^Fig. 10 and 11, respectively, show the resulting 3-D visual representations for Cs_6_AgBiCl_2_ and Cs_6_AgBiBr_2_. Notably, all other constants show large deviations from a fully spherical (3-D) shape, with the exception of linear compressibility, highlighting the anisotropic character of these materials.

Additionally, anisotropic parameters, such as Zener (*A*_Z_) and Universal anisotropic parameters (*A*_U_), have been determined using the second-order elastic constants (SOECs).^71,72^Table 3 presents the computed values for these parameters. In isotropic materials, the Zener elastic parameter, which is mainly related to shear anisotropy in materials, stays constant at 1. But any departure from unity, as we can observe in *A*_U_ and *A*_Z_ in Table 3, indicates that the material under study exhibits anisotropic behavior. The elastic constants have later been used to calculate the Kleiman parameter (*ξ*). Table 3 indicates that the materials have a higher resistance against bond angle distortions and bond bending due to the observed low value of *ξ*.

We determined the melting temperature using the following equation in order to assess their stability at high temperatures,^73^

3 *T*(*m*) = [553 + 5.911*C*_11_] ± 300

The predicted melting temperatures are determined to be 609.15 ± 300 K for Cs_6_AgBiCl_2_, 616.48 ± 300 K for Cs_6_AgBiBr_2_ and 607.08 ± 300 K for Cs_6_AgBiI_2_. Notably, the high value of the melting temperature suggests that these materials exhibit stability even under extreme temperatures.

### 3.7 Phonon dispersion and thermal properties

**Fig. 12:**
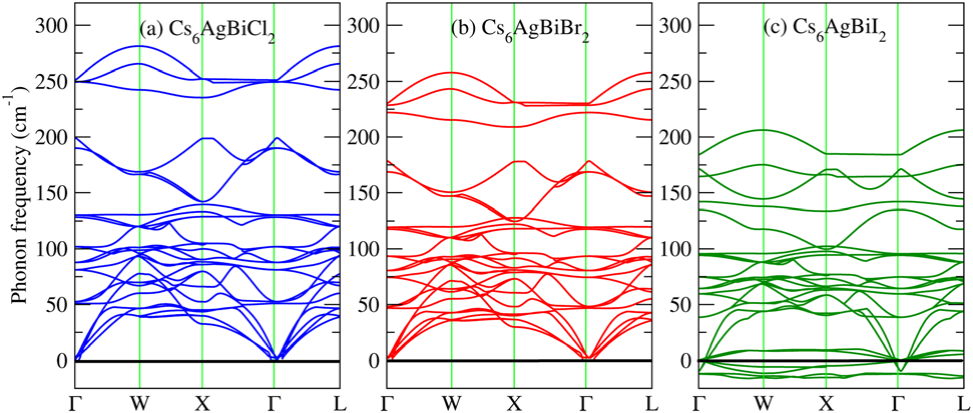
Calculated phonon band structures of Cs_6_AgBiX_2_ (X = Cl, Br, I).

To further confirm the stability, adding to the elastic constant results, we use a 2 × 2 × 2 supercell in the well -established PHONOPY package.^74,75^ The calculated phonon band structures of Cs_6_AgBiCl_2_, Cs_6_AgBiBr_2_ and Cs_6_AgBiI_2_ are shown in Fig. 12a–c. Cs_6_AgBiCl_2_ and Cs_6_AgBiBr_2_ are dynamically stable which also validates the elastic results, while Cs_6_AgBiI_2_ has negative phonon frequencies up to −25 cm^−1^. Hence, Cs_6_AgBiI_2_ has been shown to be unstable by elastic as well as vibrational analysis.

Aside from these elastic constants, Debye temperature (*Θ*_D_) is an important thermal parameter that is closely linked to several physical properties such as a compound’s melting point and specific heat capacity. It is possible to calculate Debye temperature using average sound velocity *v*_m_.^76^4
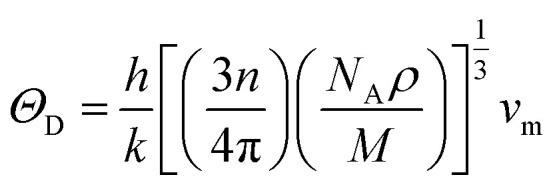
The variables *h*, *k*, *n*, *N*_A_, *ρ*, and *M* stand for Planck's constant, Boltzmann constant, atomic number, Avogadro’s number, material density, and molecular mass, respectively. The stronger the connection, the higher the Debye temperature or sound velocity. The average sound velocity in polycrystalline materials can be expressed as follows:^77^5
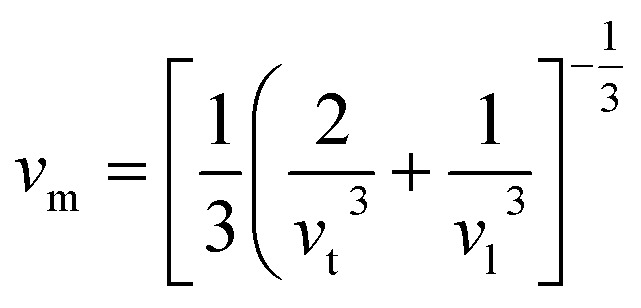
where *v*_t_ and *v*_l_ are the transverse and longitudinal sound velocities, respectively, that may be derived using Navier's equation and elastic constants (*B* and *G*).^78^6
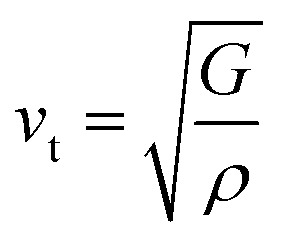
7
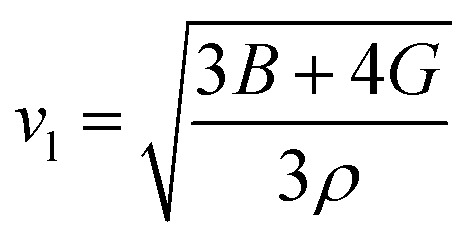


Table 4 displays the values of *v*_t_, *v*_l_, *v*_m_, and *Θ*_D_ that were derived from the aforementioned relations. Cs_6_AgBiCl_2_ and Cs_6_AgBiBr_2_ have the highest Debye temperature indicating that these are thermally more conductive. These findings also provide insight into the bond strength hierarchy.

**Table 4:** Thermal properties of Cs_6_AgBiCl_2_ and Cs_6_AgBiBr_2_

Compound	Cs_6_AgBiCl_2_	Cs_6_AgBiBr_2_
*v*_t_ (m s^−1^)	1006.9	904.04
*v*_l_ (m s^−1^)	1737.84	1670.64
*v*_m_ (m s^−1^)	1086.32	985.35
*Θ*_D_ (K)	473.6	451.3
*ω*_D_ (THz)	61.9	58.9

## 4 Conclusion

In this work, structural, electronic, optical, photocatalytic, mechanical, vibrational, and thermodynamical behaviors were studied for new double antiperovskite (DAP) Cs_6_AgBiX_2_ (X = Cl, Br, I). Band structures were calculated with and without spin orbit coupling (SOC). Using the TB-mBJ approach (hybrid) reveals bandgap values of 1.504 eV, 1.491 eV, and 1.392 eV for Cs_6_AgBiCl_2_, Cs_6_AgBiBr_2_, and Cs_6_AgBiI_2_ respectively. To gain deeper insight into the orbital contributions of chlorine (Cl), bromine (Br), and iodine (I) near the Fermi level, analysis of the total density of states (TDOS) and partial density of states (PDOS) were studied. In addition to electronic properties, the investigation extended to optical properties, including real and fictitious refractive indexes, extinction coefficients, optical conductivities, absorption coefficient, and reflectivity. The elastic and phonon properties demonstrate that Cs_6_AgBiCl_2_ and Cs_6_AgBiBr_2_ are stable except Cs_6_AgBiI_2_. The calculated optimal bandgap (∼1.5 eV) and high absorption coefficient (∼30× 10^4^ cm^−1^) of Cs_6_AgBiCl_2_ and Cs_6_AgBiBr_2_, suggest their potential for solar cell applications. In addition, our photocatalytic results suggest that these Cs_6_AgBiX_2_ have more oxidizing capacity due to good visible-light absorption capability that can be used for cheap oxygen production *via* solar water splitting.

## Data availability

Data will be made available upon request.

## Conflicts of interest

The authors declare that they have no known competing financial interests or personal relationships that could have appeared to influence the work reported in this paper.
